# American Dental Association

**Published:** 1897-11

**Authors:** 


### Reports of Society Meetings.

### AMERICAN DENTAL ASSOCIATION.

(Continued from page 660.)

*August 4, 1897.—Second day.—Morning Session.*

The meeting was called to order by President Truman at a quarter to ten o'clock.

The Secretary read the minutes of the previous session, which were approved. The Secretary also read a letter from S. B. Hartman, regretting that he cannot be in attendance, and sending his good wishes to the American Dental Association and the Southern Dental Association.

The Secretary read a resolution from the Southern Dental Association inviting all members of the American Dental Association (upon presentation of a card to be signed by Dr. Cushing) to attend clinics given by the Southern Dental Association. The invitation was received with thanks, and the Secretary was instructed to send a return invitation to the Southern Dental Association to attend our meetings and be granted the privileges of the floor.

### COMMITTEE ON CREDENTIALS AND ETHICS.

Dr. Jackson reported that the committee had audited the accounts of the Treasurer and found them correct. The committee also reported that at the last meeting charges of unprofessional conduct were preferred against Dr. Horton, of Cleveland. Dr. Horton having received notice of such charges through publications and having made no defence, the committee recommend that his name be dropped from the roll of membership. A motion was made to such effect and carried. The committee has also audited the Publication Committee's account and found it correct.

Report accepted.

*Dr. Crouse.*—According to the programme as printed, Dr. Harlan was to give the annual address this morning. He is not here, and I move that we substitute in place of that Dr. Cassidy's address.

Motion carried.

*The President.*—Dr. Harlan wrote me some weeks ago that it would be impossible for him to be at this meeting, but his letter came too late to appoint any one in his place.

Dr. Cassidy, of Covington, Ky., then read a paper entitled "Relations of Chemistry to Dentistry."

(For Dr. Cassidy's paper, see page 719.)

DISCUSSION.

*Dr. Barrett*, of Buffalo.—I should be happy to discuss this question if it were not mainly devoted to a field which I have not cultivated. I am all at sea upon the mistakes to which Dr. Cassidy has called attention. We are ambitious to be known as educated men. We claim a scientific standing, and yet some of our representative men cannot spell correctly. Sometimes the most ludicrous blunders occur in text-books and publications, and I am very glad indeed when a man of some erudition calls attention to our shortcomings. We get on the fence, flap our wings, and crow, and we do not see the insecure foundation upon which we stand. I never learn anything from the man who says I know it all and substantiates everything I say; but I do learn from the man who shows me that I am mistaken, who tells me I am wrong, and proves it. I shall read the paper and make a study of it.

*Dr. Peirce*, of Philadelphia.—I wish to express my great pleasure in the paper. It was instructive to me, and one which is true in its scientific conclusions.

*Dr. St. George Elliott*, of New York.—I would like to ask Dr. Cassidy what his views are in regard to Dr. Black's statement. The Institute of Stomatology, of New York, sent out circulars to a large number of dentists within the past few weeks, asking their views on Dr. Black's statement that the density of a tooth has nothing to do with its tendency to decay. The answers have not yet come in, but I would like Dr. Cassidy's view.

*Dr. Cassidy.*—It seems to me we know nothing about the density of a tooth. It is the specific gravity, compared to the weight of the tooth; therefore the density has nothing to do with the decay. The hardness and compactness may, of course, have something to do with it. If Dr. Black meant density in its true meaning, of course he is correct.

*Dr. Elliott.*—Dr. Black states distinctly that in speaking of density, he did not consider the hardness to cutting instruments. That seems to explain the whole thing.

*Dr. Taft.*—I am very glad and proud that we have men in our profession who can consider these subjects as the author of this paper did. Papers like this enter upon the marrow and pith of the subject involved, and I think this body would do well to encourage such efforts by any aid in their power.

*Dr. Smith.*—I can only commend the paper. Those of us who know Professor Cassidy, know that in making a public statement or reading a paper, he is careful to be on solid ground. He spoke of the manner in which cement fillings disintegrate at the cervical border. We often hear at meetings of associations that cement fillings stand just as well at the cervical border as they do anywhere in the cavity. Dr. Cassidy accounts for the dissolution at that point by the development of the alkaline reaction. If that be correct, all the statements made by those who make permanent fillings which stand at the cervical borders and under the gum tissue must be very far wrong. Only lately a gentleman said that if his fillings failed at all, it was away from those borders. We have a development of ammonia, and that will dissolve any cement filling.

*Dr. Patterson.*—We know the dread with which students in colleges approach the study of chemistry. Why it is so I can scarcely tell, unless it is that we have no dental teachers of chemistry. It has been found by me that those students who come into our colleges as graduates in pharmacy are the best students we have. They make splendid dentists, and we are glad to accept them; and when the National Association of Faculties gave the graduates in pharmacy credit for one year they did a good thing. I would like to say a word in regard to the disintegration of oxyphosphate fillings at the cervical border. If it is proved by experimentation that those fillings will and do disintegrate from alkalinity at that portion of the filling, then Dr. Smith's statement and that of Dr. Cassidy may be substantiated. I have made many experiments along that line, and I will say that they do not, if properly mixed in alkaline solutions, disintegrate at that border or any other border perceptibly. If you can do out of the mouth what you do in the mouth, any alkaline solution that is found in the mouth will not affect an oxyphosphate filling properly made. I am not a chemist myself. The only thing I can say is, what I have tried with acid and alkaline solutions, and if you will try it you will come to the same conclusions practically as I have done.

*Dr. Smith.*—I did not expect to make this issue, but I doubt very much if you can produce the conditions outside of the mouth that you get in the mouth. I have made my best efforts with that material, and the portion that rises above the cervical edge will be intact when the other is destroyed, because we have deposited at that line the foods, which in fermenting produce an alkaline reaction. The conditions at the cervical border are such that fermentation takes place. Those conditions cannot be produced out of the mouth, and a test out of the mouth would not be proper. Let me appeal to every member present if it is not true that many times, if the filling fails at all, it is at the cervical border. We can give no assurance of permanency, especially in a mouth which is neglected, and where we invariably have a reaction.

*Dr. Barrett.*—Dr. Smith says the fermentation is an alkaline one.

*Dr. Smith.*—I should have said putrefactive one.

*Dr. Cassidy.*—That is a distinction without a difference, as I understand it. Putrefaction is an alkaline fermentation, while what we call fermentation is of an acid nature. Vinous fermentation is also of an acid nature. All forms of fermentation, as we restrict the term, mean that we end with an acid,—acetic acid, etc. Putrefaction is used to indicate fermentation of nitrogenous matters which produce ammonia and its derivatives. Dr. Patterson says he has experimented out of the mouth with oxyphosphate cements. So have I; but the actions that occur in the mouth prove certain facts. If we acknowledge, as we must, that ammonia is developed by alkaline fermentation or putrefactive fermentation, we must acknowledge that ammonia is developed in the mouth, and I must say that oxyphosphate filling, prepared carefully, placed in a tooth out of the mouth in a dry condition, and allowed to stand in a dry place over night, if placed in a five-per-cent. solution of common ammonia in water (which is really about a one-five-thousandth solution), the next morning you will find the filling has all disintegrated, the oxide of zinc will be a precipitate, and phosphoric acid will have united with the ammonia. If we acknowledge that ammonia is developed in the mouth, this will take place in oxyphosphate fillings.

*Dr. Barrett.*—I am glad to have the line drawn so closely between putrefaction and fermentation. Putrefaction is destructive fermentation with the evolution of offensive products, due to the nitrogenous product which is present. While both are essentially the same products, they are different classes; still, they belong to the fungi family, while inorganic products are of a different character. I am glad Professor Cassidy explained the difference between the two, for there has been a great deal of confusion in regard to it.

*Dr. Smith.*—I would like Professor Cassidy to make a little more clear why it is that these fillings disintegrate more at the border than at the other points. Why do we have more fermentation at that point?

*Dr. Cassidy.*—The finishing of the filling has a great deal to do with it. When a filling is finished under the margin of the gum and a little roughness or imperfection is allowed there, which is more apt to be the case at that point than elsewhere, it sets up irritation and inflammation of the soft part of the gum or any of the tissues where inflammation occurs. Inflammation is concomitant with the process of fermentation called putrefaction, and the characteristic or typical ending of putrefaction is ammonia or some of its derivatives. The ptomaines, for instance, are derivatives of ammonia, and develop at that point probably more by the inflammation than by the *débris* that might be lodged there.

*Dr. Barrett.*—I call attention to a white, cheesy deposit, with which, no doubt, Professor Cassidy is familiar. That seems to account for the thing. It is found at the gingival margin upon the teeth.

Dr. Peirce moved that, as Dr. Case is obliged to leave, Section I. take precedence of Section VI., and Dr. Case read his paper next. The motion was carried, and Dr. Case proceeded to read his paper.

*Dr. Case.*—Part of the paper that I am about to present was read at the last meeting of the Illinois State Dental Society, especially the part that refers to the fundamental principles of force. As explained at that time, I have continued the paper to its present state. My object and desire in presenting it here is to give it before as large a number of dentists as possible. It has reference largely to the teaching of orthodontia, and I hope to receive a fair discussion and criticism of its merits, that I may be able to eliminate the errors which it may contain.

An abstract of Dr. Case's paper follows:

### PRINCIPLES OF FORCE AND ANCHORAGE IN THE MOVEMENT OF THE TEETH.

BY C. S. CASE, D.D.S., M.D., CHICAGO, ILL.

If there is one thing more important than another in the science of regulating teeth, it is a mind that is well trained in the simple laws of physics, with the ability to practically apply these laws to the invention, construction, and management of regulating appliances.

In the voluminous literature and teachings upon the subject of orthodontia and dentistry in general there is little to be learned of these most important basic principles. In our endeavor to become a great profession and completely dissociate ourselves as professional men from the fearful calamity of being classed as tradesmen and mechanics, there has been an unfortunate tendency to underrate the value of certain branches of knowledge that lie at the very foundation of dentistry, and which should form the only true basis to scientific training for almost everything we undertake as dentists.

In my teachings I dwell at considerable length upon the laws which govern force and the mechanical advantages of different methods of applying it in the practical movement of teeth. I treat a regulating apparatus, together with the teeth to which it is attached, as a machine. The best definition of a machine is that it is an instrument introduced between a moving force and a resistance with the view of changing the direction of the force or otherwise modifying it. In that branch of my teachings entitled "Construction of Regulating Appliances, and their Management," I endeavor to arrange the work systematically, according to the distinct mechanical principles involved in the scientific application of the required force. I divide the whole subject under this head into two general divisions, entitled respectively "Action" and "Reaction." Under the head of "Action," which pertains exclusively to the movement of malposed teeth to the direct action of force, I take up, first, the movement of crowns in every direction; second, the movement of roots in every direction, with or without the movement of their crowns; third, rotating or turning the teeth on their own axes; fourth, intrusion and extrusion, or the gradual forcing of teeth in or out of their sockets.

The second main division of the work, entitled "Reaction," pertains to the management of the opposing force,—action. This I divide into, first, reciprocating or movable anchorages, describing methods for utilizing the force of reaction in the movement of other malposed teeth, and, second, stationary or static anchorage appliances. I do not mean by this that I confine the teachings of this work to a single branch at a time, to the exclusion of all others, but I endeavor to blend them into each other, illustrating the principles as I progress with practical cases.

In contemplating the construction of a molar anchorage appliance that will permit the least possible movement of the included teeth, the principal object should be to so construct the device that the great tendency of tipping of the crowns will be prevented. If this be fully accomplished and the tooth or teeth are held in an upright position, the applied force will be evenly distributed over the entire anterior or posterior surfaces of the alveoli for all the roots, increasing the stability of the anchorage to an incalculable degree. If the appliance is loosely attached to the teeth or permits the slightest hinge-movement, as would arise from a removable attachment or a single uncemented band that encircles two or more teeth, there would be nothing to prevent this tipping tendency, though such an anchorage might be sufficient for many purposes, if it is attached to a sufficient number of teeth, and the applied power is always less than their combined natural inertia. An instance frequently arises in the regulation of teeth where it is eminently desirable to obtain an anchorage of the greatest possible stability. When considerable force is required to reduce an irregularity, two or three teeth should be included in the grasp of the anchorage appliance. The addition of a second tooth to the anchorage will not only double its stability but incalculably increase it by the support which the two teeth can be made to give to each other by proper construction of the appliance.

In its construction, German silver or platinized gold bands should be selected that are six- to ten-thousandths of an inch in thickness (or Nos. 34 to 30 B. & S. gauge) and as wide as the teeth will permit. When these are soldered and properly contoured to perfectly fit the crowns, take a plaster impression of them in position, after which carefully remove the bands, place them in the impression, and fill with Teague's or any good investing material. This will hold them in proper relative position during the soldering process. Solder should be flowed between the bands, uniting their approximal surfaces and filling the V-shaped spaces on every side. To more perfectly reinforce the stability of the appliance, fit and solder to the lingual surfaces a flattened piece of No. 16 wire, after which the tube or tubes are to be attached for the power bars or traction wires for the movement of the anterior teeth. In attaching the tubes the advantage of applying the power as near to the gingival margin as possible should be remembered. When such an appliance is fitted and cemented to the teeth, it will hold them rigidly in its grasp, in an upright position. If the bands are thin and narrow or the apparatus is not sufficiently reinforced by the solder and otherwise, the slight yielding of the material under great strain will allow the teeth to tip and soon break loose from their attachments.

Instances frequently arise where only one tooth can be used for an anchorage on one or both sides of the mouth. These teeth, not being supported by adjoining teeth, will readily tip if not properly sustained. In fact, a molar tooth that is allowed to tip will offer but little more resistance to force than a bicuspid, but if sustained in an upright position, it will be impossible to force it bodily through the process with any ordinary power. This is especially true of the lower molars, which, when tipped to an anterior or posterior inclination, are readily forced from their embedment or partially lifted out of their sockets; but if held in an upright position, the two broad roots will resist immovably a large amount of force. When a single isolated molar is used for an anchorage attachment, the band should be wide and thick, fitted and cemented as carefully as a crown, with rigid attachments for inflexible extensions. However perfect the band and its attachments, if a flexible traction wire is used to transfer the power no obstruction is offered to the tipping tendency of the molar. The same is true with an inflexible power-rod, if the band is thin, narrow, and yielding, or in any way movable upon the tooth, or if the power-tube is short and loosely fitted to the rod.

DISCUSSION.

*Dr. Jackson.*—I have been very much interested in the paper. The doctor has been describing to us his way of teaching his method of anchorage, but he has not shown any specially new features. My system of anchorage is so entirely different that I think I shall let the gentlemen who have more general ideas of anchorage speak on the subject. I would not wedge the teeth apart to put two thicknesses of collar between them to gain an anchorage. That anchorage need not necessarily excite a congestion or inflammation that would work detrimentally, but I do not approve of the practice. There are several methods besides what I have described of gaining a perfect anchorage attached to molars. One that I have used for several years is making a cap over the molars and bicuspids, if they are not to be moved, and contouring it more accurately than a collar could be, because the grinding surface, being a little larger than the neck, it is very difficult to cement perfectly a collar on a tooth that is tapering towards the gum. Many make the mistake of not seeing that the collars are firmly cemented. In the system that I speak of, we would cement a piece of very thin metal, fortified by a thicker piece of metal, and then cement it firmly to the tooth. If the molar is not fully erupted, it can be made to assist in the anchorage by a thin, flat piece of metal soldered to the cap, and then cementing the whole firmly to the tooth.

That is probably one of the best methods we have of insuring an anchorage that will not permit the teeth to tip forward or backward. I appreciate the principle the doctor has intended to give us, from the scientific description of the use of the lever, which we should all understand thoroughly before attempting to regulate the teeth. I have but very little difficulty, in the method I practise, of the movement of the teeth detrimentally out of their natural position, when used for anchorage, because the first thing I do is to determine what influences are going to be exerted and to adopt methods that will prevent that movement. It is certainly irrational to believe that we can move several teeth any distance with a single tooth on either side of the arch as anchorage. We must fortify that anchorage. I get great benefit from the use of the plate, for instance, in contracting the anterior part of the arch, although Dr. Case has discarded the use of the plate entirely.

*Dr. Noble*, of Washington.—I have been intensely interested for years in the principles which have been enunciated so clearly and distinctly in the paper this morning. No one should undertake the correction of irregularities until those mechanical principles are thoroughly understood, and then he should study each and every case by careful impressions before undertaking to apply the principles. I am an enthusiastic admirer of what is known as the Jackson system. There has never been a time in my practice of over thirty years that I have not had cases of regulating going on, and therefore I speak somewhat from experience. I have come to the conclusion that the Jackson method will cover a larger number of cases with a greater simplicity than we have ever obtained before. I doubt very much if the general profession understand yet the simplicity of that system. If they did study and understand it as I have seen it illustrated in cases of my own within the past few years, I am sure they would use it. The principles are all correct. I say it without fear, and I believe that I can substantiate it with models that I have, that nearly all of these cases can be accomplished with a much more simple apparatus than those illustrated here to-day, however correct they may be. I indorse the principles that Dr. Case has given us, and I shall be very glad to show the models I have brought, and my way of accomplishing those ends, to any gentleman who wishes to see them.

*Dr. McKellops.*—This is a question that deserves a great deal of attention from my profession. Many men make a mistake when they undertake to do this thing, and that has ruined more mouths than we probably are aware of. I have met this gentleman at different meetings, and the results he accomplishes are wonderful, by understanding the anatomy of the jaws and the face. The profession ought to give this strict attention. We know the amount of crooked faces and jaws that come to us, and now under the experienced hands of such men a great deal of good can be accomplished.

*Dr. Rich.*—This paper is a very important one in the advance of this part of our profession. For the first time in any exhibition I have ever witnessed has a person endeavored to show us an apparatus for regulating the teeth in which the correct mechanical principles have been clearly stated and illustrated. The great fault of all the methods that have been presented for regulating teeth is that there has never accompanied them a correct exposition of the proper mechanical principles that should govern the construction of these apparatuses. Here is, for the first time in my experience, a system presented which is founded on correct mechanical principles which no one can deny, and it illustrates how they can be applied with the least amount of force to the portions of teeth that are to sustain the parts to be moved.

*Dr. Ottolengui,* of New York.—I rise with some reluctance to discuss this paper, because of the fact that this is my first year as a member of your Association. Ever since these theories were first advocated, and this exceedingly ingenious method of regulating was introduced, I have hoped for the opportunity of discussing this matter with the author of the apparatus present. I have preferred to do that rather than appear in print in opposition to some of the theories, because I recognize the fact that I may be entirely in error, and the exchange of erroneous views only encumbers the literature on the subject; whereas, if I express views now which the author can refute, the refutation will appear in print in the same article with my antagonism of the principle. In the first place, admitting the marvellous ingenuity, and giving the author due credit for the conception of his work, I do not conceive the general applicability of it which he claims. I think he said it was almost unlimited in its applicability. If by that he means it can be applied to a tremendous variety of cases, I concede that; but if he means to a tremendous variety of patients, I deny it. The use of abutments which are movable and which must be sustained in their position by the extreme ingenuity which the author has shown, is in itself an admission of weakness in the system. If we must depend on a lot of posts in the mud to move another post in the mud, that is a fundamental error. A common error which has been made in the past in using this sort of anchorage, to which we are often driven, is that we have depended on the anterior anchorage. So far as I know, one of the earliest to publish a method to solve this problem was Dr. Jackson. By somewhat different principles the same result is obtained. Dr. Jackson often depends on the anterior teeth for anchorage, and also the molar teeth to move intermediate teeth. Whenever I have been called upon to get a third attachment,—to find a fixed portion in the anterior part of the mouth by placing a band in the mouth and uniting it with wire to the two posts in the back of the mouth,—the first difficulty that has met me is the sacrifice of the invaluable space that is occupied by the bands in the front of the mouth. This method depends upon many bands and the sacrifice of a great amount of space, all of which, if I were regulating the teeth, I would feel bound to close up. The author seems not to mind leaving spaces between the teeth, because he shows us a fixed appliance which is to retain his teeth.

Coming back to a further argument against the general applicability of this method, we find that the whole power of resistance to the movement which is to be made must come from the posterior teeth which are to support bands. Since this was promulgated I have had between twenty and thirty cases of protruded arches pass through my hands, and I have not found one case in which the posterior teeth offered a sufficient anchorage to the required power which would be needed to move the anterior teeth backward; that is why I am unwilling to wait until a child is grown up and all of its bones are hardened. I prefer to begin the retraction of a jaw as early as I find it,—the ninth year, if possible,—before the canines have appeared. I prefer to take the chance of taking the canines back with the unerupted processes, while the bones are soft, and I cannot do it with this method. In some of these illustrations we find that the anchorage has been sustained by both molars, which would indicate the fourteenth or fifteenth year, or an early development of the teeth in that arch. I must admit that there are exceptions that occur. A child is to come to me in the fall who is only eleven years of age, but whose twelfth-year molars are developed. The rarity of these cases, however, places them among the curiosities. I frequently find in these mouths very tiny lateral incisors and central incisors, teeth which are conical in shape, which does not allow you to use the band, so that the theory is not applicable as frequently in our hands as it seems to have been in the case of the children of the West.

I want to say a word about this other fixture. We have an arrangement by which the solitary anchor-tooth is to be held. What will be the result if it is to be prevented from tipping? Without going into an extended examination of the fundamental physical principle involved, we have the molars fastened so that they cannot tip, and we have a force which is supposed to carry back eight or nine teeth on the strength of the buccal power of the teeth in the back, which has been further weakened by the removal of a tooth in front, leaving a boneless socket. Why should not those teeth go forward bodily? I say they will do so. The contour of the face is not affected by the moving of one tooth. By utilizing the anchorage of the posterior teeth for the resistance to our force, we are undermining and weakening the very thing upon which we most rely. I have seen teeth come to me with the retainer accurately fitting and the protrusion returning, and yet the dentists have said the teeth are all right because the retainer fits. So it does; but the necessity which says we must have a retainer recognizes the tendency of the front teeth to return; that force requires a fixed resistance, and if it is not great enough, they will move forward, retainer and all. Consequently, a method which is so old that we relegate it to the past, can be used, and that is utilizing the back of the head for anchorage and moving the teeth in that way.

*Dr. Guilford.*—I am one of those who have always admired the work of Dr. Case, and I am glad that we have in the field of orthodontia so able a workman. He has done much excellent and original work in this line, and we must give him credit for doing work which no one has done before him. If they have thought of it, they have not shown it. The movement by which he holds one portion of the tooth while he moves the other is beautifully shown. He has been the first one to do it, and he deserves our everlasting gratitude for it. To-day, while he has shown us the fundamental principles, I think he has made one or two errors. Dr. Ottolengui has anticipated me a little. In regard to the matter of levers, I do not think those are illustrations of levers at all. A lever must have a fulcrum somewhere, and those have not; they are simply illustrations of raising a weight. The other point I have spoken to Dr. Case about before. In a case like this, when you make an extension from a band upward and you apply your power either on the outside or the inside, you get your power where the base of the rod is. You gain something by having a wire to attach the bow wire, but that is all. I object to the claim that you get greater power; it is a convenience, nothing more. This bar would have as much effect as the one shown above.

Dr. Case showed us a wide band around the tooth; he seemed to feel that you would get an advantage by going beyond the gum. You get only so much as is attached to the band; going beyond it gives no advantage whatever.

In regard to some of the other points, we all recognize as an important matter the retaining and counteracting power applied in one direction by power applied in another direction. In regard to this matter of the posts, he explained to us why it was that in moving a post of that kind up to a certain point you had movement of the tissues nearest the surface. In the movement of a tooth of this kind, we must consider the outer layer of the bone, which is very dense. If we wanted to tip this root in the direction in which it has been tipped, we would want to take advantage of that layer. I do not doubt but what Dr. Case does that where he wants to move it back bodily, but he did not mention it.

Dr. Case lays great stress upon the fitting of the bands and burnishing them closer to the tooth. I believe in a band not occupying any more space than it should; but when you get one that adapts itself very closely to the tooth, you do not have much room for the cement, and it is the cement, as Dr. Jackson says, which holds the band there. I do not care to fit my bands so closely, but rather depend upon the cement to hold them in place. Dr. Ottolengui mentioned the putting of so many bands upon different teeth, which takes up the interdental space. If you want to move them inward, and you put so many bands on, you come to a point where these bands occupy space that you want. It is easy to do that in many cases. If I had a number of teeth that I wanted to move in, I would, instead of putting four bands, put only two.

In the matter of molars, if we have one band here and another one adjoining it (illustrating), it is a very difficult matter to press those teeth aside, so as to get room for your double bands; but a very simple way to do it is to get a little space, and making your band in the shape of a horseshoe, you only have one thickness instead of two. Dr. Jackson spoke of swedging a cap. That is a most excellent way in deciduous teeth; but in the permanent teeth often occlusion of the opposite teeth will prevent it, and therefore I prefer bands in the majority of cases. For the beauty of the appliance and the beautiful construction, as shown in the models, I have the greatest admiration for Dr. Case. He deserves all the credit we can give him for the work he has presented.

*Dr. Case.*—I met with about the usual reception that I expected somewhat, and I am quite surprised. I am not surprised at some of the errors that Dr. Ottolengui made; but when Dr. Guilford says certain things that I would expect only from some of my students, I am exceedingly surprised. As I have but a little time, I would like to explain the simple little point of the relation of the two forces. Before I do so, permit me to say that I thank the gentlemen very much who have spoken favorably of me and my methods. My paper was brought here for the purpose of establishing, as far as possible, some fundamental principles relative to teaching students in the regulation of teeth. What have we been doing for the last fifteen years on that line? Why is it that this special department is far behind everything else? Simply because men have come before conventions of this kind with their models, showing how they succeeded in certain cases, and have brought the models to show the methods employed. I want to establish some foundation principle upon which this thing rests, that we may arrive at a more scientific and perfect position in this branch of orthodontia.

In regard to the method of applying these forces, the gentlemen say they do not see any difference between the two methods; that this bar extending above the gingival margin of the gum applies no different force, simply because it comes down and makes its attachment to the same place. One of the laws of force is that the impelling power is always in the direction of the force. If it is a rigid bar, it is impossible for many students to understand how that applies a force that is in direct line with the force on the root of that tooth. Suppose you are trying to move a tooth, the root of which is embedded in a mass of material. The resistance to that is between points along the posterior surface of the alveolus. If we can apply a force that is between the two points, we would carry the entire root in this direction in an upright position.

*Dr. Rich.*—Your theory is that that band must be rigid with the tooth itself?

*Dr. Case.*—Yes; and if that could be extended as high as the end of the root, if the bar is part of it and perfectly rigid, it is the same as applying the force to the end of the root.

*Dr. Grant Molyneaux.*—Dr. Case is wrong when he says it makes no difference how high you extend the bar. If you apply power to the lever directly at right angles to the power you are moving, it would bring the power a little higher on the root than if the power were directed straight across the gum margin. If this power is directed in this line, the power will be exerted parallel with it. If it is exerted directly across, it will intersect on the other side of the body to be moved. If the bar extends the other way, the tooth will be drawn in the opposite way.

*Dr. Case.*—Suppose you have a small tooth, and you make a rigid bar and apply the force at this point, would it not tip that tooth over? If you make it rigid, you will find it is exactly equal to applying the force of the upright bar.

Subject passed.

The organization of sections then took place, after which the meeting adjourned.

(To be continued.)

